# Large Incarcerated Inguinal Hernia: A Case Report

**DOI:** 10.7759/cureus.27822

**Published:** 2022-08-09

**Authors:** Jonathan D Lutchka, Chase W Morrison, Andranig A Adjemian, Paul D Walker

**Affiliations:** 1 Anatomical Sciences, Wayne State University School of Medicine, Detroit, USA; 2 Ophthalmology, Visual and Anatomical Sciences, Wayne State University School of Medicine, Detroit, USA

**Keywords:** scrotal swelling, left indirect inguinal hernia, cadaver dissection, cadaver case report, indirect inguinal hernia

## Abstract

A 90-year-old white male cadaver was found to have an incarcerated left inguinal hernia (IH). Although IHs are a very common pathology, the size and extent of this IH make it a unique case study. Upon gross dissection of the abdominal and pelvic cavities, 79 cm of small and large bowel was removed from the scrotal sac. The extent of the herniation had enlarged the scrotal sac to over 14 cm in both height and width and over 10 cm in depth. The herniation also caused the penis to become buried in the skin and not visible.

## Introduction

Inguinal hernias (IHs) are a common pathology where contents from the peritoneal cavity migrate inferiorly towards the inguinal canal. IHs are divided into different categories based on the location of the migration. The two most common are direct and indirect. Direct IHs migrate medially to the inferior epigastric vessels in Hesselbach's triangle. Indirect IHs, which are more common, migrate laterally to the inferior epigastric vessels and push through the deep inguinal ring to gain access to the inguinal canal. Once herniations enter the inguinal canal, they can migrate further inferiorly into the scrotal sac, where they can cause significant pain and discomfort. Some hernias, such as the one explored in this case, are classified as incarcerated or irreducible. This refers to hernias that cannot be put back in place by external manipulation.

IHs are thought to have both a congenital and acquired component resulting in a bimodal distribution, peaking with children five years of age and with adults over 70 years of age, and are more common in males (90%) than females (10%) [1]. The lifetime incidence of IHs in men is nearly 25%. The cause of an IH is a weakened area of the abdominal wall, which allows for the passage of peritoneal contents through it. Some diseases affecting connective tissue, such as Ehlers-Danlos syndrome and Marfan syndrome, have been noted to contribute to IHs. With adult patients, increases in intra-abdominal pressure due to obesity, chronic cough, chronic obstructive pulmonary disease (COPD), heavy lifting, or straining can also play a role in IH development [1]. Symptoms of an IH may appear gradually over time or have a sudden onset. Defects present at birth that contribute to an increased risk of an IH may not be detectable until symptoms begin [2]. Asymptomatic patients with IHs can experience accidental discovery during routine physical exams. Symptomatic patients have varying manifestations, including bulging and groin pain. Physical activity and coughing can elicit worsened symptoms, oftentimes with a burning or pinching sensation. Pain caused by IHs can radiate inferiorly into the scrotum or down the leg. IHs classified by incarceration or strangulation can present severe pain or obstructive symptoms involving herniated contents. A physical exam is generally sufficient for diagnosis. Imaging, although seldom used, can help diagnose a hernia or provide more information as to the type of hernia.

Hernias, especially those that become incarcerated, can be problematic for the patient. If left untreated, they can progress to become strangulated hernias, leading to bowel ischemia and fatal outcomes. Most patients with incarcerated hernias must undergo emergency surgery within 24 hours to reduce the risk of bowel necrosis, yet they still experience high mortality rates [3]. In patients with incarcerated hernias, roughly 15% require bowel resection due to strangulation and ischemia of the bowels [4]. Another rare complication that has been reported is the torsion of the greater omentum which can cause severe acute abdominal pain [5].

## Case presentation

A 90-year-old white male cadaver was selected for pedagogic dissection as part of the Gross Anatomy Laboratory prosection program completed by rising second-year medical students at Wayne State University School of Medicine in Detroit, Michigan. The cause of death in this man was incarcerated IH, small bowel obstruction, septic shock, and acute hypoxic encephalopathy, as indicated by the pathology report. During gross dissection, abnormal anatomy of the peritoneal cavity was observed, notably the size and extent of the incarcerated IH on the left side.

Gross examination

**Figure 1:**
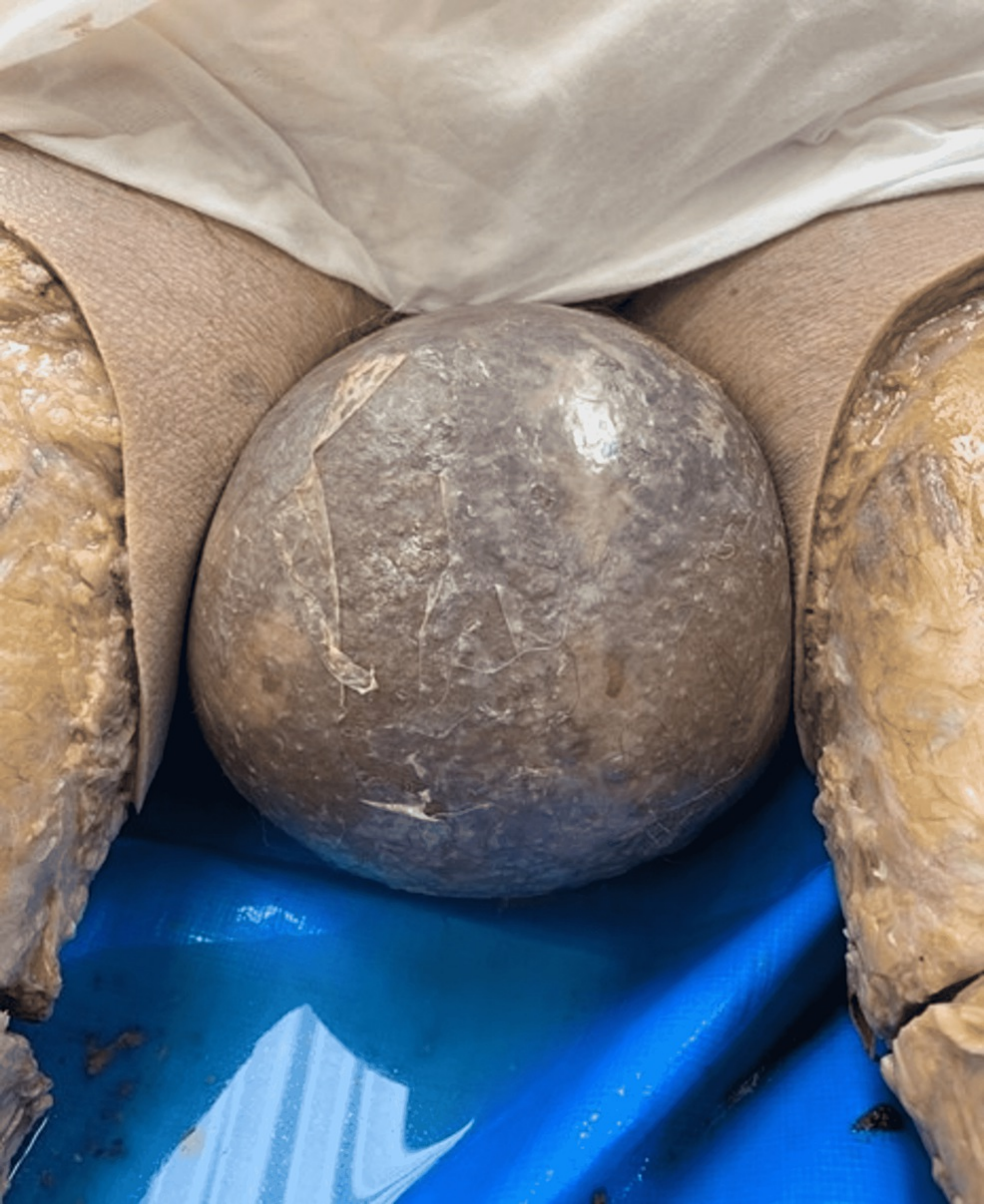
Gross view of enlarged scrotal sac upon examination.

In-situ examination revealed a grossly enlarged scrotal sac. The overlying skin was discolored and began to break down (Figure 1).

**Figure 2:**
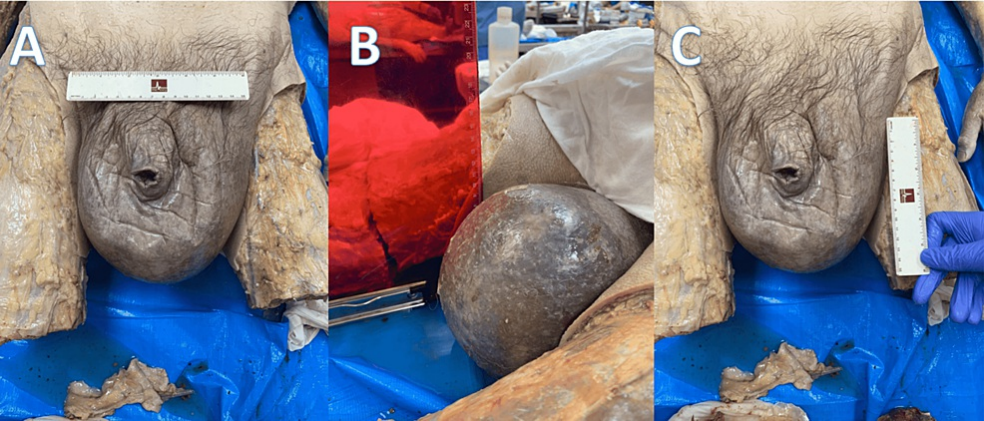
Photographs depicting scrotal sac measurements (A) width of 15 cm at the base of the penis, (B) depth of 11 cm measured anterior to posterior (cadaver in prone position), (C) height of 14 cm measured from the base of the penis.

The scrotal sac was roughly 15 cm in width (Figure 2A), 11 cm from anterior to posterior (Figure 2B), and 14 cm in height (measured from the base of the penis) (Figure 2C). The penis was no longer visible due to the swelling in the area.

**Figure 3:**
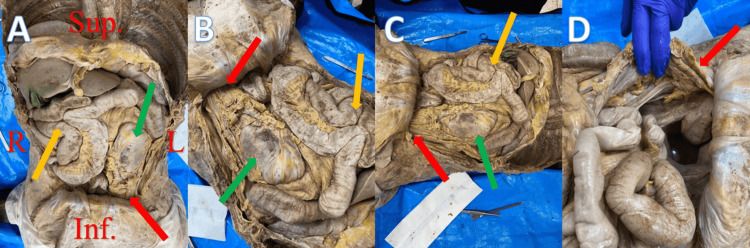
Photographs illustrating the abnormal anatomical position of abdominal contents. All red arrows in 3A-3D depict peritoneal contents exiting the peritoneal cavity. All green and orange arrows in 3A-3C depict stomach and small intestine, respectively. (A) anterior view of the abdomen in anatomical position; (B) anterosuperior view (hernia contents entering deep inguinal ring on left); (C) left-sided anterolateral view; (D) right-sided anterolateral view with herniated bowel reflected laterally.

The abdominal contents were displaced from the normal anatomical position upon opening the peritoneal cavity. The stomach was pulled inferolaterally towards the left (green arrows in Figure 3A-3C), the large intestine was not visible, and only a small portion of the greater omentum was visible (Figure 3). Most of the jejunum and ileum were shifted superolaterally towards the right (orange arrows in Figure 3A-3C), while the duodenum seemed to stay close to its normal position. The liver, gallbladder, spleen, pancreas, and rectum were all in normal positions. Herniated peritoneal contents were observed to be exiting the peritoneal cavity via the left deep inguinal ring (red arrows in Figure 3A-3D).

**Figure 4:**
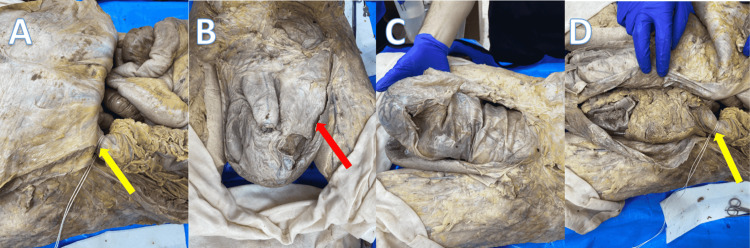
Photographs depicting (A) position where herniated contents were tied (yellow arrow); (B) position of scrotal sac incision (red arrow); (C) contents of hernia with peritoneum intact and scrotum opened; (D) contents of hernia with peritoneum reflected laterally (yellow arrow showing the same tie as Figure 4A).

The contents of the herniation were tied off where they entered and exited the deep inguinal ring for visualization (Figure 4A, see yellow arrow). Next, the scrotal sac was cut to reveal the herniated peritoneal membrane (Figure 4B, see red arrow). The cut opening of the peritoneal cavity was then extended inferiorly down the scrotum to visualize the contents of the herniated peritoneal membrane (Figure 4C-4D).

The contents of the herniated peritoneal membrane appeared to include approximately ½ of the transverse colon, the splenic flexure, the totality of the descending and sigmoid colon, and a portion of the greater omentum. The involvement of the greater omentum in the hernia resulted in the stomach being pulled inferolaterally towards the left deep inguinal ring, but remained in the peritoneal cavity. The total length of the small and large intestines was measured to be 463 centimeters. The portion involved in the herniation was measured to be 79 centimeters, roughly 17% of the length of the intestines.

The herniation also appeared to push the left testicle inferiorly against the floor of the scrotal sac. Upon gross dissection of the left testicle, it was noted to be difficult to find any intact sections of the left spermatic cord. Further dissection of the pelvic region revealed pelvic mesh on the right side, suggestive of an operative procedure for a right-sided IH. The mesh extended from the medial border of the deep inguinal ring to approximately the midline of the pubic symphysis and extended posteroinferiorly down the anterior wall of the pelvic cavity for about 5 centimeters.

## Discussion

IHs are a common pathology in the United States [1]. However, the size and extent of the IH found in this study make it unique. A separate case report of an IH similar in size noted that a wait-and-see approach is often adopted for large IHs such as the one in this case due to the size and complexity. However, the risk of waiting can set the stage for other complications and therefore recommends aggressive treatment, if possible [6].

IH repair is relatively common, with over 20 million inguinal herniorrhaphies performed every year worldwide. IHs can typically be diagnosed by physical examination and are more commonly found in males. Common risk factors for IH include family history, previous contra-lateral hernia, age, abnormal collagen metabolism, prostatectomy, and low BMI. A standard repair technique has not yet been established for groin hernias; however, several procedures are available, including Lichtenstein tension-free repair, laparoscopic transabdominal preperitoneal repair (TAPP), totally extraperitoneal repair (TEP), Shouldice, and others [7]. The use of mesh has also been shown to significantly reduce the reoperation rate following hernia repair [8].

In this case, the reward of aggressive surgical treatment and the risk of life-threatening surgical complications must be weighed. Because we did not have access to full medical history, it is challenging to make a definitive choice postmortem about what should have or could have been done to treat this patient. Emergency IH surgery is associated with significantly higher morbidity and mortality than elective surgical repair of IH (SRIH) in high-risk geriatric patients. Elective hernia repair in these patients should be considered to reduce the risk of the need for intestinal resection and the length of hospital stay [9]. However, it is essential to note that due to the patient's age and other likely causes of death, he would have been at high risk for postoperative complications, a longer hospital stay, and increased morbidity with an elective hernia repair [10].

It is important as a physician to balance the risks and benefits to achieve medically successful treatment with the social and emotional well-being of the patient. It is in the clinicians' hands to provide safe and effective treatments that they believe are in the patient's best interest without providing overly assertive guidance on the best decision for the patient's personal goals for treatment [11].

## Conclusions

Because of the limited information and medical history given about the cadaver due to confidentiality, only speculations can be made about the causes of the IH and reasons for abstaining from treatments. Due to this patient's age, it is a common scenario that a period of constipation could have increased intrabdominal pressure enough to 'open the door' through a weakened abdominal wall. Once the IH was present, there were many possibilities as to why it was not treated. This patient could have been at the end of life, where comfort is a higher priority than surgical intervention. Depending on how long the IH took to reach its final size, the potential feeling of embarrassment of letting the IH develop to this point could have deterred this patient from seeking treatment. Lastly, because of the evidence of other surgical interventions we found in this patient, such as lumbar and cervical spinal fusions, as well as surgical mesh in his right pelvis, we think that socioeconomic barriers were less likely to prevent him from receiving care, but still could have been a possibility at this point in his life.
